# Isolated Aphasia From a Left Parietotemporal Infarct Secondary to a Patent Foramen Ovale: A Case Report

**DOI:** 10.7759/cureus.84678

**Published:** 2025-05-23

**Authors:** Aamir Shaikh, Sabahuddin Hajjar, Shahnawaz Notta, Hezborn Magacha, Venkata Vedantam

**Affiliations:** 1 Department of Internal Medicine, James H. Quillen College of Medicine, East Tennessee State University, Johnson City, USA

**Keywords:** cardioembolic stroke, cryptogenic stroke, isolated aphasia, middle cerebral artery, patent foramen ovale

## Abstract

Isolated aphasia (IA) as the sole manifestation of stroke is rare and is often attributed to metabolic, toxic, or psychiatric disorders. Without typical stroke symptoms, such as hemiplegia or cranial nerve deficits (CND), IA can be easily overlooked. We present the case of a 41-year-old male accountant with a history of hyperlipidemia who presented to the emergency department with the sudden onset of an inability to read or speak, accompanied by confusion and anxiety. On examination, he had no CNDs, and his strength, sensation, reflexes, and cerebellar function were normal. His vital signs, blood glucose, and initial computed tomography (CT) scan were unremarkable, and his National Institute of Health Stroke Scale (NIHSS) score was 3. The patient declined tissue plasminogen activator (tPA) therapy. Computed tomography angiography (CTA) showed no intracranial stenosis, but magnetic resonance imaging (MRI) confirmed an acute infarct in the left parietal and temporal lobes within the middle cerebral artery (MCA) distribution. Further evaluation with a bubble study during echocardiography revealed a right-to-left interatrial shunt consistent with a patent foramen ovale (PFO). He was started on dual antiplatelet therapy, a statin, and speech therapy. Neurology and cardiology specialists recommended outpatient PFO closure and placement of an implanted loop recorder for arrhythmia monitoring. At discharge, the patient showed a significant improvement in his aphasia, though occasional recurrences persisted. This case highlights a rare presentation of ischemic stroke with IA, a condition that is more often attributed to stroke mimics such as metabolic or functional causes. Comprehensive neurological and cardiovascular evaluations are essential, particularly given the strong association between IA and cardioembolic sources such as PFO. Early multidisciplinary intervention is crucial to optimize outcomes, as this case illustrates the importance of identifying and addressing cryptogenic causes of IA to prevent recurrent strokes.

## Introduction

Aphasia, a language disorder resulting from damage to portions of the brain responsible for language, is a common and devastating cognitive impairment in stroke patients. However, isolated aphasia - aphasia without accompanying symptoms such as dysarthria, visual-field defects, or motor or sensory deficits - is distinctly rare, occurring in only about 5.1% of stroke cases [1]. This presentation can complicate diagnosis and treatment, as it may not immediately suggest a stroke to clinicians.

Isolated aphasia can manifest in various neurological disorders such as stroke, seizure, multiple sclerosis, Creutzfeldt-Jakob Disease (CJD), and hyperglycemia [2-5]. For example, isolated aphasia occurs as the primary symptom in approximately 1% of sporadic CJD cases, a rare prion disease where such a presentation is atypical [4,6]. It can also be a manifestation of seizure-related stroke mimics, with only a few reported cases in the literature [2].

Stroke often presents with various symptoms, including speech disturbances and hemiparesis. While dysarthria, characterized by slurred or slow speech, is more prevalent, aphasia remains a critical concern due to its impact on communication abilities [7]. In the absence of common stroke mimics, isolated aphasia may point to an underlying cardiogenic cause.

A notable association exists between isolated aphasia and atrial fibrillation (AF), highlighting the potential for this symptom to act as an independent marker for cardiogenic sources of embolism [1]. Similarly, a patent foramen ovale (PFO), a congenital heart anomaly characterized by an opening between the left and right atria, can facilitate the passage of emboli, potentially leading to strokes [8]. While a PFO is often asymptomatic, it is notably associated with cryptogenic stroke, particularly in younger populations without traditional cerebrovascular risk factors [9]. The detection of PFO in patients with cryptogenic stroke, especially those presenting with isolated aphasia, emphasizes the need for thorough cardiovascular evaluation, even in the absence of typical stroke symptoms.

In this context, we present a unique case of a 41-year-old male who visited the emergency department with symptoms of slurred speech, confusion, receptive and expressive aphasia, but without hemiplegia, and with a National Institute of Health Stroke Scale (NIHSS) score of 3. An initial computed tomography angiography (CTA) revealed no intracranial stenosis or occlusion but indicated diminished enhancement in the left parietal lobe. Magnetic resonance imaging (MRI) confirmed an acute infarct in the left parietal and temporal lobes of the middle cerebral artery (MCA) division. A subsequent echocardiogram with a bubble study showed an interatrial right-to-left shunt with conclusive evidence of a PFO. This case underscores the diagnostic complexity and significance of identifying PFO in patients presenting with stroke characterized by isolated aphasia.

## Case presentation

**Figure 1:**
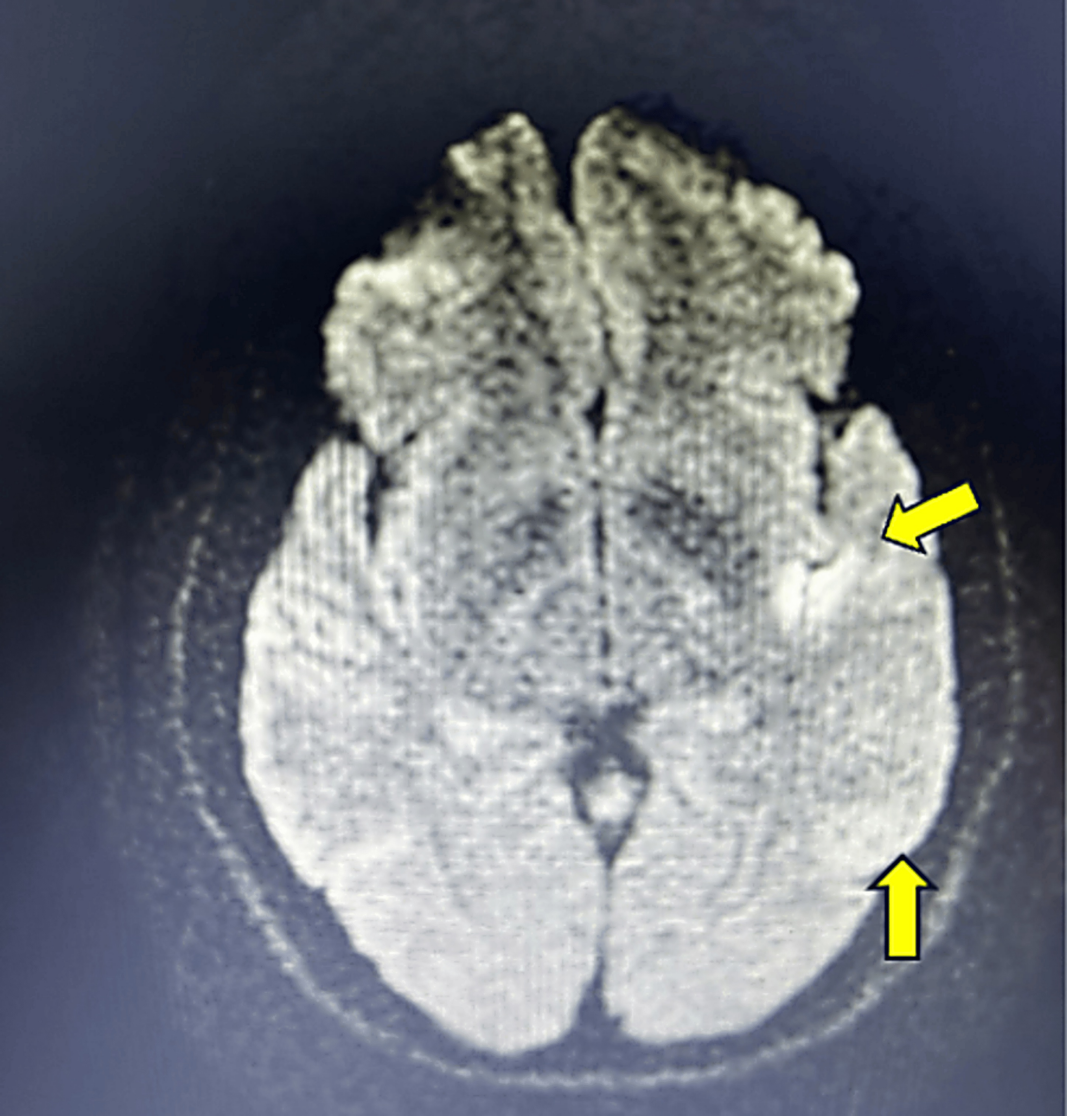
A T2-weighted MRI revealed a hyperintense signal consistent with acute infarcts in the left temporal and parietal regions

A 41-year-old male with a history of hyperlipidemia presented to the emergency department with sudden-onset receptive and expressive aphasia, accompanied by brief confusion and transient vision changes. These additional symptoms resolved shortly after arrival, leaving isolated aphasia as the primary persistent neurological deficit. He declined tissue plasminogen activator (tPA) despite partial early improvement. Initial vital signs included a temperature of 97.8 °F, heart rate of 64 bpm, blood pressure of 146/100 mmHg, respiratory rate of 18 breaths per minute, and oxygen saturation of 97% on room air. His NIHSS score was 3. A CTA of the head and neck revealed no evidence of intracranial stenosis or occlusion but raised concerns about diminished enhancement in the left parietal lobe. MRI confirmed an acute infarct in the left parietal and temporal lobes within the middle cerebral artery (MCA) territory (Figure 1).

**Figure 2:**
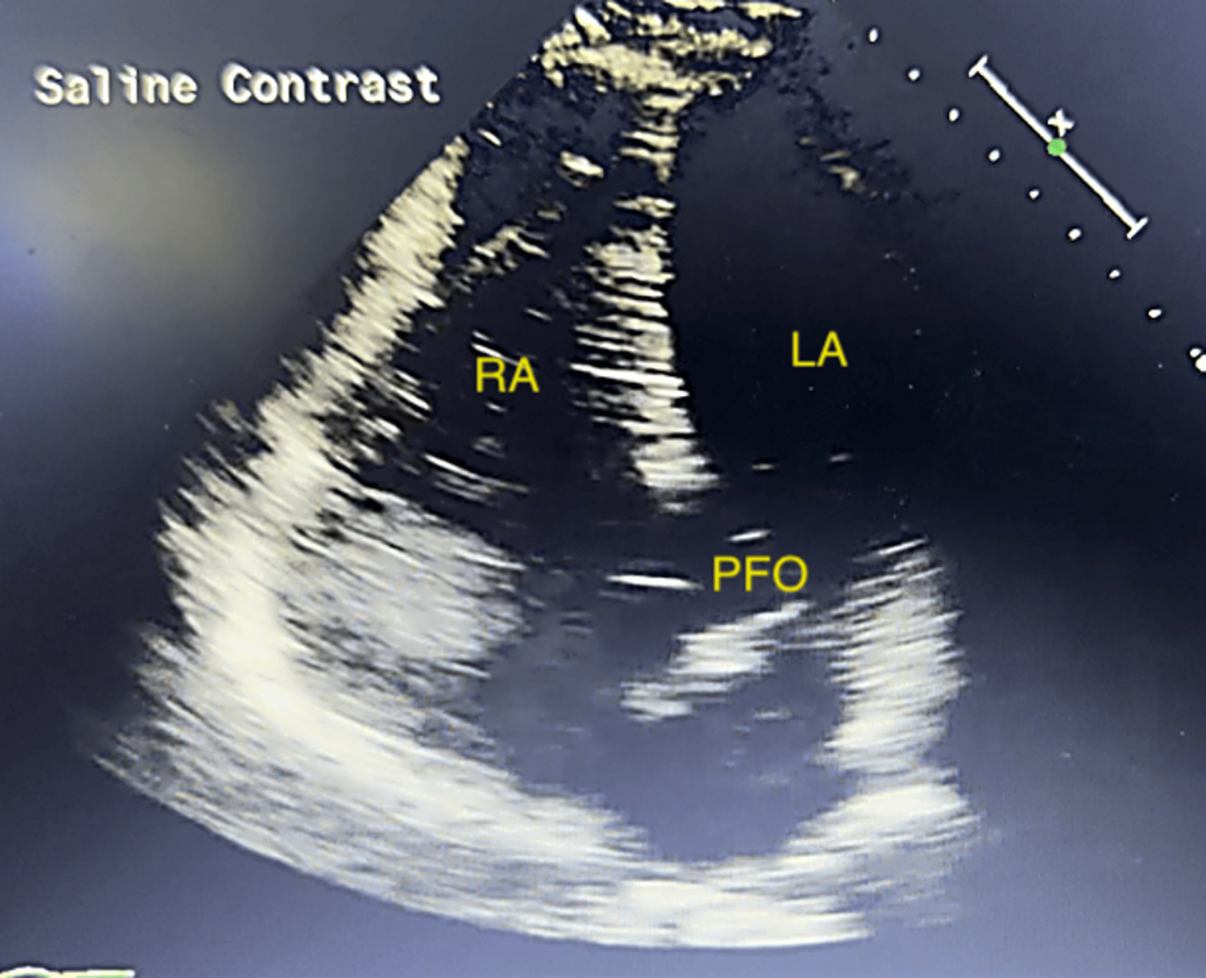
Transthoracic echo with bubble study showing contrast shunting through the patent foramen ovale

The patient was started on aspirin, clopidogrel, and atorvastatin, and the neurology team was consulted. An electrocardiogram (EKG) showed no acute changes compared to previous studies. Laboratory evaluations, including a complete blood count (CBC), comprehensive metabolic panel (CMP), drug screen, and glucose levels, were unremarkable. A carotid ultrasound did not reveal significant stenosis. Further evaluation with transthoracic echocardiography and a bubble study identified a right-to-left interatrial shunt, consistent with a PFO (Figure 2). Cardiology was consulted and recommended outpatient PFO closure. Neurology suggested continuing dual antiplatelet therapy and statin therapy, pursuing PFO closure, and monitoring for arrhythmias with an implantable loop recorder.

On hospital day three, the patient was discharged on dual antiplatelet therapy, a statin, and a plan for outpatient PFO closure, along with the placement of a LINQ implantable loop recorder (Medtronic, Ireland) for long-term cardiac rhythm monitoring. A hypercoagulability workup was performed, yielding results within normal limits. The patient was ultimately diagnosed with a rare ischemic stroke involving the left parietal and temporal lobes, attributed to a paradoxical embolism from an undiagnosed PFO. At discharge, the patient demonstrated significant improvement in his symptoms but continued to experience mild receptive and expressive aphasia.

## Discussion

The present case of a 41-year-old male with isolated aphasia secondary to an acute infarct emphasizes the clinical challenge and management complexities associated with this rare stroke presentation. Isolated aphasia, which occurs in only about 5% of acute stroke cases [1], requires a nuanced approach to diagnosis and treatment, particularly when the underlying cause is a cardioembolic event such as a PFO.

Management strategies for isolated aphasia involve a multidisciplinary approach, incorporating speech and language therapies, cognitive neurorehabilitation, and thorough identification of the underlying cardioembolic mechanisms [10]. Given that isolated aphasia can result from cardioembolic sources, such as AF or PFO, patients should undergo careful cardiac screening to distinguish between presumed cardioembolic transient ischemic attack (TIA) or stroke. Differentiating these conditions is crucial, as secondary prevention strategies vary depending on the embolic source [1]. Thrombolysis has also been demonstrated as a safe and effective treatment for patients with isolated aphasia, even those with low NIHSS scores, supporting its use in acute management [11].

In cases where PFO is identified as the stroke's etiology, the decision to close the PFO becomes pertinent. While recent trials such as CLOSE (Patent Foramen Ovale Closure or Anticoagulants Versus Antiplatelet Therapy to Prevent Stroke Recurrence), REDUCE (GORE® Septal Occluder Device for Patent Foramen Ovale (PFO) Closure in Stroke Patients), and RESPECT (Randomized Evaluation of Recurrent Stroke Comparing PFO Closure to Established Current Standard of Care Treatment) have shown that PFO closure can reduce recurrent stroke rates in cryptogenic stroke patients [12], the role of PFO closure in patients with atypical stroke presentations like isolated aphasia is less clear. The potential benefit of PFO closure must be weighed against procedural risks, especially in cases with unique presentations like isolated aphasia [13].

Further complicating management, isolated aphasia requires targeted rehabilitation strategies. Emerging therapies, such as self-managed computerized speech and language therapy, have shown significant promise in improving outcomes for chronic stroke aphasia patients, enhancing their participation in recovery [14]. Additionally, innovative approaches like repetitive transcranial magnetic stimulation (rTMS) and music therapy accompanied by transcranial direct current stimulation are being explored to address language dysfunctions in post-stroke aphasia [15,16].

Future research should focus on refining the management pathways for patients with isolated aphasia, particularly those with cardioembolic sources. Effectively detecting atrial heart disease and other cardiovascular conditions is essential for preventing recurrent strokes and improving outcomes post-cardioembolic events [17]. Additionally, the potential role of left atrial appendage (LAA) closure should be investigated in patients with stroke of suspected cardioembolic origin [12].

In conclusion, isolated aphasia post-stroke presents unique diagnostic and therapeutic challenges, particularly when linked to cardioembolic sources like PFO. A comprehensive, multidisciplinary approach is essential for optimizing outcomes, and future studies should focus on improving detection and management strategies for this rare and complex condition.

## Conclusions

Clinicians should recognize isolated aphasia as a potential early indicator of cardioembolic stroke, particularly in patients with atrial fibrillation (AF) or patent foramen ovale (PFO). Despite its rarity, isolated aphasia warrants thorough cardiovascular evaluation, including consideration of PFO closure in appropriate cases, to prevent recurrent strokes. Prompt identification and treatment, including the use of thrombolysis and targeted rehabilitation strategies, are essential for optimizing patient outcomes. Maintaining vigilance for isolated aphasia can lead to more accurate diagnosis and effective management of underlying cardioembolic conditions, ultimately improving clinical care.
